# Editorial: Molecular basis of stem cells underlying plant vitality

**DOI:** 10.3389/fpls.2024.1371814

**Published:** 2024-02-07

**Authors:** Chunli Chen, Alfredo Cruz-Ramirez, Yonghong Zhang, Masaki Ishikawa

**Affiliations:** ^1^ National Key Laboratory for Germplasm Innovation & Utilization of Horticultural Crops, Hubei Hongshan Laboratory, College of Life Science and Technology, Huazhong Agricultural University, Wuhan, China; ^2^ Molecular & Developmental Complexity Group, Unit of Advanced Genomics, LANGEBIO-CINVESTAV, Irapuato, Mexico; ^3^ Hubei Key Laboratory of Embryonic Stem Cell Research, School of Basic Medicine, Hubei University of Medicine, Shiyan, China; ^4^ Division of Evolutionary Biology, National Institute for Basic Biology, Okazaki, Japan

**Keywords:** plant stem cell, stem cells signaling, stress response, regulatory network, plant regeneration

Plant stem cells persisting in tissues throughout the plants life are proliferative and undifferentiated cells and are crucial for growth and development. In embryogenesis, the fertilized egg cell, a totipotent stem cell, provides the cells that form the seedling. Post-embryonically, pluripotent stem cells inhabit niches where they divide asymmetrically to self-renew and to generate daughter cells, forming diverse plant tissues. Indeed, the adaptation capability of the plants towards environmental challenges relies on the preservation of plant stem cell niches. Moreover, physical damage often induces *de novo* formation of the stem cell niches in differentiated tissues, making it possible to regenerate organs from wounded parts or an entire plant body from a dissected tissue. Given their crucial roles in plant development and regeneration, it is imperative to elucidate the pivotal roles of stem cells on plant vitality at the molecular level. The research advancements, such as single-cell RNA sequencing, CRISPR-Case9 gene editing, live cell imaging, optical coherence tomography, bioinformatics and computational modeling, 3D printing and tissue engineering, and single-cell cloning techniques have contributed to study the roles of stem cells in plant development and stress response.

A number of key factors crucial for the stem cell maintenance and homeostasis have been identified in plants, with ongoing efforts to clarify their regulatory systems. Notably, the homeodomain transcription factor WUSCHEL (WUS) and the class I KNOX transcription factor SHOOT MERSTEMLESS (STM) play essential roles in preserving pluripotent stem cells within the shoot apical meristem. WUS functions in the WUS-CLAVATA (WUS-CLV) pathway (Heidstra and Sabatini, 2014; Ma et al., 2019), a system crucial for maintaining the balance between self-renewal and differentiation of stem cells. STM, expressed throughout the shoot meristem, represses stem cell differentiation. These two transcription factors have been shown to interact, playing a significant role in the regulation of stem cell homeostasis. An AP2-family transcription factor PLETHORA (PLT), recognized as a master regulator in the root meristem, has been also shown to regulate *de novo* shoot regeneration (Kareem et al., 2015). Another transcriptional factor, a key regulator in regeneration rapidly induced upon wounding, is WOUND INDUCED DEDIFFERENATION 1 (WIND1) (Iwase et al., 2011). Additionally, the plant hormones, including auxin, cytokinin, jasmonic acid, and gibberellins, environmental signals, light (in transmitting light signals to regulate plant growth), and temperature (affecting cell division and differentiation) are also marked as significant factors in stem cell homeostasis. Recent studies have shown that JA levels are elevated upon injury, and the expression of ERF109 and ERF115 was induced, but still, the role of JA is considered situation-dependent as JA has also been reported to inhibit callus formation after hypocotyl cutting (Ikeuchi et al., 2017; Zhang et al., 2019). Therefore, the underlying molecular events behind cell cycle activation and stem cell fate transition remain elusive.

This Research Topic aimed to explore the plant stem cells characteristics through the molecular basis of stem cells underlying plant vitality and the mechanisms underpinning the maintenance of pluripotency and genome homeostasis. The role of a zinc-finger transcription factor KNUCKLES (KNU) in floral meristem activity was studied to explore its early expression and broader influence beyond the *WUS* expression domain. It contributes to the broader knowledge of plant stem cell maintenance and plant floral development (Kwaśniewska et al.). Significant insights are provided into the complex role of CLAVATA3/EMBRYO SURROUNDING REGION-RELATED (CLE) 16/17 peptides in root development, showing their dual function in stem cell differential and lateral root emergence and their interaction with different molecular pathways (Zhang et al.). The formation of new meristems in plants involves a dynamic and transient phase, the pre-meristem, marked by unique regulatory networks; this phase is crucial for the subsequent establishment of a functional stem cell niche and overall development of plants meristem (Nicolas and Laufs). The study shed light on the complex mechanisms by which plants react to changing environments through dynamic body formation by transcriptional regulators. This process is crucial for the regulation of gene expression in response to various external stimuli, demonstrating a sophisticated level of environmental responsiveness in plants (Burkart et al.). Meristem genes, particularly STM and its related pathways, are crucial for the maintenance of stem cells in SAM and floral meristems in Arabidopsis, which is co-opted in *K. pinnata* for sexual reproduction. Another research study sheds light on the genetic mechanisms underlying vegetative reproduction in Kalanchoe and the role of meristem genes in this process (Jacome-Blasquez and Kim). New insight is further provided into how ABA signaling impacts stem cell fate in moss by altering cell division, cell polarity, and cell cycle, leading to the formation of stress-resistant stem cells. This research enhances the understanding of molecular mechanisms behind the tradeoff between growth and stress response in plants (Beier et al.).

Under the umbrella of the United Nations Sustainable Development Goals (SDGs), a profound understanding of plant longevity and vitality is pivotal in addressing global challenges, especially those concerning food safety and environment preservation. In particular, the understanding of the mechanisms underlying the regeneration and sustainability of the plant stem cells under stress, during growth and throughout development is vital for ensuring a stable food supply for both current and future generations. The available information on master transcription factors for stem cell regulation provides fragmentary pictures of molecular networks governing plant vitality. Integrating spatiotemporal phytohormone control and epigenetic regulation into these networks at the cellular level could enhance our understanding of the fundamental principles underlying plant stem cell regulation. To further explore these aspects, beyond current plant biotechnology, it will inevitablly need employing innovative techniques including higher resolution imaging, multi-omics at the single-cell level, and spatiotemporal precise gene editing, and utilize analytical and computational tools.

## Author contributions

CC: Writing – original draft. AC: Writing – review & editing. YZ: Writing – review & editing. MI: Writing – review & editing.
